# Prediction of sudden cardiac death using artificial intelligence: Current status and future directions

**DOI:** 10.1016/j.hrthm.2024.09.003

**Published:** 2024-09-06

**Authors:** Maarten Z.H. Kolk, Samuel Ruipérez-Campillo, Arthur A.M. Wilde, Reinoud E. Knops, Sanjiv M. Narayan, Fleur V.Y. Tjong

**Affiliations:** 1 Department of Clinical and Experimental Cardiology, Amsterdam UMC Location University of Amsterdam, Heart Center, Amsterdam, The Netherlands; 2 Amsterdam Cardiovascular Sciences, Heart Failure & Arrhythmias, Amsterdam UMC location AMC, Amsterdam, The Netherlands; 3 Department of Computer Science, ETH Zurich, Zurich, Switzerland; 4 Department of Medicine and Cardiovascular Institute, Stanford University, Stanford, California

**Keywords:** Artificial intelligence, Deep learning, Implantable cardioverter-defibrillator, Machine learning, Sudden cardiac death, Ventricular arrhythmia

## Abstract

Sudden cardiac death (SCD) remains a pressing health issue, affecting hundreds of thousands each year globally. The heterogeneity among people who suffer a SCD, ranging from individuals with severe heart failure to seemingly healthy individuals, poses a significant challenge for effective risk assessment. Conventional risk stratification, which primarily relies on left ventricular ejection fraction, has resulted in only modest efficacy of implantable cardioverter-defibrillators for SCD prevention. In response, artificial intelligence (AI) holds promise for personalized SCD risk prediction and tailoring preventive strategies to the unique profiles of individual patients. Machine and deep learning algorithms have the capability to learn intricate nonlinear patterns between complex data and defined end points, and leverage these to identify subtle indicators and predictors of SCD that may not be apparent through traditional statistical analysis. However, despite the potential of AI to improve SCD risk stratification, there are important limitations that need to be addressed. We aim to provide an overview of the current state-of-the-art of AI prediction models for SCD, highlight the opportunities for these models in clinical practice, and identify the key challenges hindering widespread adoption.

## A. Scope of the problem

**Figure 1:**
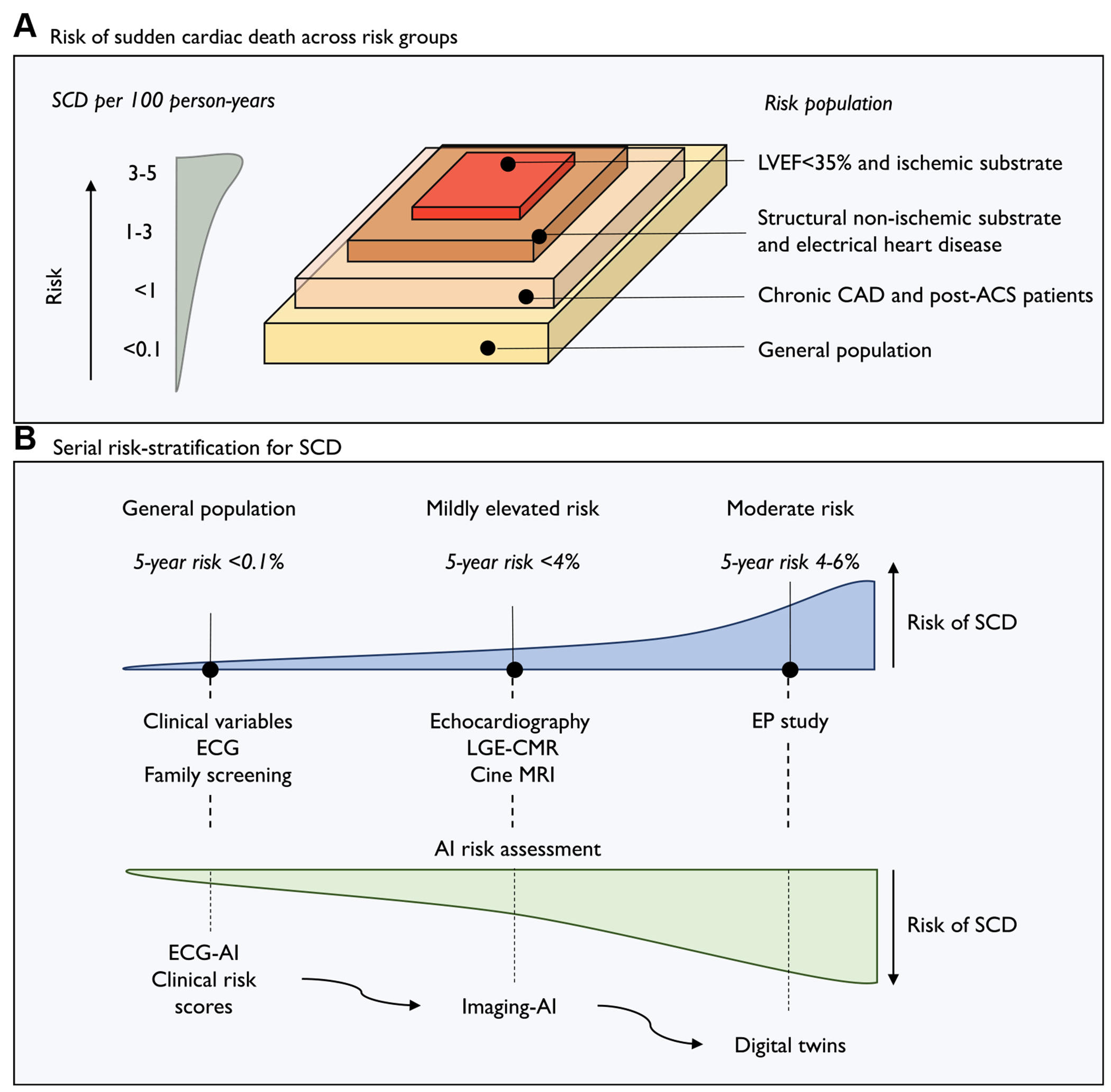
Risk of SCD across the general population and at-risk populations. **A:** The relative risk of SCD is highest among patients with a history of cardiac disease or genetic predisposition. However, the absolute number of SCD cases is greater in the general population. Models developed from specific cohorts or clinical repositories with defined phenotypes may be less accurate when applied to broader populations with different SCD risk profiles. Accurate assessment of the balance between arrhythmic and nonarrhythmic mortality risks at the individual patient level is essential. **B:** A serial testing strategy has been proposed for SCD screening. This approach uses algorithms tailored to the diagnostic modalities realistically available within a given target population, prioritizing individuals for more detailed and resource-intensive screening, such as imaging or invasive testing. ACS = acute coronary syndrome; AI = artificial intelligence; CAD = coronary artery disease; CMR = cardiac magnetic resonance; ECG = electrocardiography; EP = electrophysiology; LGE = late gadolinium enhancement; LVEF = left ventricular ejection fraction; MRI = magnetic resonance imaging; SCD = sudden cardiac death.

Sudden cardiac death (SCD) remains a major public health problem worldwide, with ~250,000 cases in Europe and ~360,000 cases in the United States every year.^1,2^ SCD accounts for ~50% of all cardiovascular deaths and 10%–15% of all deaths, with up to half of the cases being the first manifestation of underlying cardiac disease.^3,4^ The incidence of SCD in the general population varies between 0.011 and 0.091 per 100 patient-years, with rates differing by sex, age group, and geographic location.^2,3,5–7^ While the incidence of SCD declined from 1990 to 2010, it has remained stable in more recent years.^2,8^ In addition, despite significant investments by the medical and research communities over the past decades, the prognosis of out-of-hospital cardiac arrest continues to remain poor, even for those who are immediately resuscitated from out-of-hospital cardiac arrest.^9,10^ This challenge is compounded by the fact that people who suffer a SCD form a heterogeneous population, including patients without a prior diagnosis of heart disease, those with severe cardiac dysfunction, and individuals with a genetic predisposition.^3,7,11^ Although the relative risk is highest among patients with a history of cardiac disease or a genetic predisposition, the absolute number of SCD cases is higher in the general population (Figure 1A).^11^

About half of the SCD cases are attributable to ventricular tachycardia (VT) or ventricular fibrillation.^1^ Long-term primary prevention of SCD using an implantable cardioverter-defibrillator (ICD) is recommended in patients with symptomatic heart failure and reduced left ventricular ejection fraction (LVEF) in the setting of coronary artery disease (CAD) (class 1a) or dilated cardiomyopathy (class 2a).^4^ Pivotal ICD studies published between 1996 and 2005, which enrolled patients with ischemic and nonischemic causes of systolic heart failure, reported incidences of SCD between 7.0 and 17.2 per 100 patient-years.^12^ However, a review by Shen et al^13^ of large heart failure trials published until 2014 noted a 55% decline in the risk of SCD over time, which could be attributed to the introduction of cardiac resynchronization therapy and new pharmacotherapies for heart failure. This reduction in SCD risk was also seen in the later ICD and heart failure trials—that is, Danish Study to Assess the Efficacy of ICDs in Patients with Non-ischemic Systolic Heart Failure on Mortality (DANISH), Dutch outcome in implantable cardioverter-defibrillator therapy (DO-IT), European Comparative Effectiveness Research to Assess the Use of Primary ProphylacTic Implantable Cardioverter Defibrillators (EU-CERT-ICD), Dapagliflozin and Prevention of Adverse Outcomes in Heart Failure (DAPA-HF), and Prospective Comparison of ARNI with ACEI to Determine Impact on Global Mortality and Morbidity in Heart Failure (PARADIGM-HF)—with incidences ranging between 1.5 and 3.3 per 100 patient-years.^14–17^ Consequently, the number of patients who receive appropriate ICD therapy has become relatively small and the majority of those with an ICD do not derive significant benefit from the device.^18^ In addition, ~70% of SCDs occur in patients who fall outside guideline recommendations and thus have not been identified as high risk before the event.^19^ Therefore, because of the competing risk from other modes of death, patients who will benefit from ICD therapy the most are not those with the highest absolute risk of SCD, but rather those with the highest risk of arrhythmic death relative to nonarrhythmic death.^18^

## B. One-size-fits-all vs personalized SCD risk stratification

There is a need for personalized tools for arrhythmic risk stratification that could guide preventive measures. Various clinical risk scores have been developed over the past years to aid early detection of individuals at risk of SCD in the general population, including the Atherosclerosis Risk in Communities (ARIC score), and patients with a preexisting risk of SCD, such as the Seattle Proportional Risk Model, VF Risk Score (VFRisk), and Multicenter Automatic Defibrillator Implantation (MADIT-ICD) scores.^18,20–23^ However, the low incidence of SCD might make mass screening for SCD risk unfeasible, as it would result in a high number of falsepositive predictions despite reasonable discriminative performance.^4,20,24^ In addition, factors that portend a higher risk of SCD increase this risk only marginally for a given individual.^24^ The burgeoning field of artificial intelligence (AI) presents new opportunities to overcome the limitations that traditional SCD risk prediction scores are affected by and move beyond the one-size-fits-all approach. AI encompasses a variety of techniques, such as machine learning (ML), deep learning (DL), computer vision, and natural language processing, that typically enable systems to carry out tasks requiring human cognition. These tasks can be divided into unsupervised and supervised methods; while supervised methods rely on labeled data that the model is trained to predict, unsupervised models operate without explicit guidance on the expected outcomes. ML typically involves algorithms that empirically “learn” patterns from manually selected (“engineered”) features, for example, a set of echocardiography measurements, which may not be immediately apparent through traditional statistical methods. Common supervised ML techniques include decision trees, support vector machines, and random forests. DL approaches, on the contrary, consists of multiple layers of interconnected neurons that form networks capable of learning representations directly from data without the need for manually engineered feature extraction. DL models are particularly effective for tasks involving complex, high-dimensional data, such as raw imaging, electrogram signals, or large-scale genomic data sets. The application of DL has shown significant promise in cardiac electrophysiology, with a noticeable shift from the more traditional ML techniques to DL methods in recent years. Advancements in DL have introduced several innovative techniques such as convolutional neural networks, generative adversarial networks, variational autoencoders, probabilistic diffusion models, or transformers. The application of such DL models, in particular convolutional neural networks and variational autoencoders, has seen rapid growth and popularity in arrhythmia research, exemplified by the automated estimation of LVEF through electrocardiography (ECG),^25^ the prediction of atrial fibrillation from wearable devices,^26^ or the detection of noise within electrograms transmitted by cardiac implanted devices.^27^ In the following sections, we present an overview of the current state-of-the-art of AI, including multimodal and dynamic models, for predicting SCD.

## C. ECG-AI for SCD prediction

Twelve-lead ECG has high potential as noninvasive screening modality for evaluating arrhythmic risk because of low cost and widespread availability.^28^ Indices from ECG reflect the 3 main mechanistic concepts of ventricular arrhythmia pathogenesis: cardiac autonomic abnormality (eg, heart rate variability), abnormal ventricular repolarization (eg, corrected QT interval, Tpeak-Tend interval, and T-wave alternans), and abnormal myocardial substrate and conduction properties (eg, left ventricular hypertrophy and QRS fragmentation).^11^ A proposed electrical risk score comprising heart rate, prolonged corrected QT interval, Tpeak-Tend interval, QRS-T angle, left ventricular hypertrophy, and delayed QRS transition zone accurately predicted SCD within a community-based cohort over an average follow-up period of 2 years, achieving a concordance index (C index) of ~0.74.^29,30^

**Figure 2:**
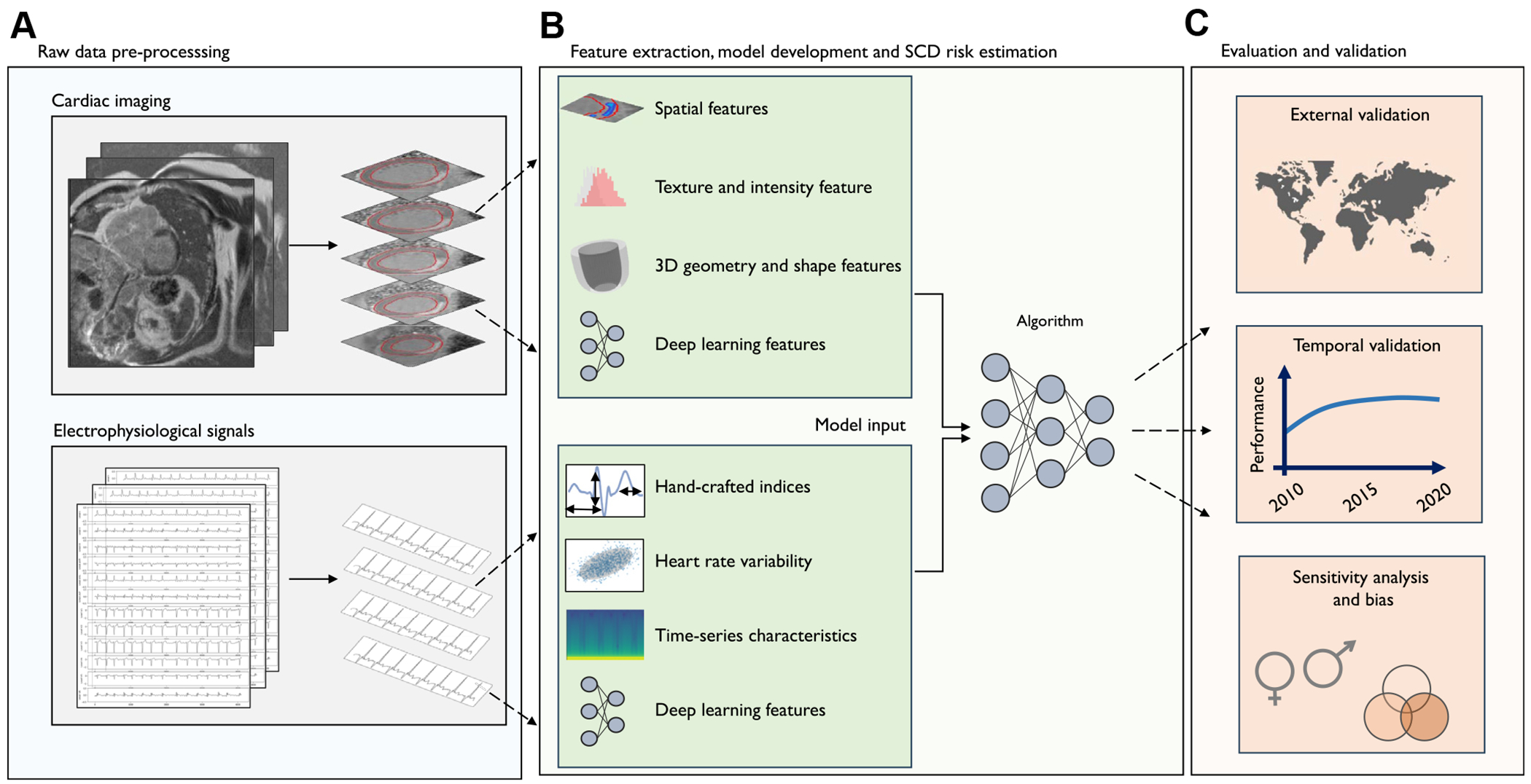
Workflow of the artificial intelligence–driven prediction model development. Example of an artificial intelligence–driven workflow for developing a risk prediction model for SCD. **A:** Input data are preprocessed to ensure effective feature extraction. This process includes data cleaning (such as handling missing values and reducing noise), transformation (including normalization, scaling, and dimensionality reduction), augmentation, and splitting (assigning cases to training, validation, and test sets). **B:** Features are extracted using either manually engineered algorithms or neural networks that learn latent representations from the data. **C:** Model performance is validated and assessed across various locations, time periods, and subpopulations. 3D = 3-dimensional; SCD = sudden cardiac death.

AI models have emerged as alternatives to these traditional risk scores by automating the extraction of features from signals, time series, or images that most contribute to a relevant clinical or physiological end point. Feature extraction is key to interpreting, summarizing, and understanding input data as a set of variables and have enabled the discovery of novel signatures that may not be appreciated by experts.^28^ Feature extraction can be categorized into 2 main approaches: those that rely on domain knowledge and human-defined rules and those that use DL to automate the process by learning hierarchical representations of features directly from raw data (Figure 2). The first approach, manually engineered feature extraction, is hypothesis based and includes standard ECG measurements, indices related to heart rate variability (time-domain, frequency-domain, and nonlinear), and mathematical time-series characteristics such as wavelet or Fourier transform.^31–33^ Mathematical indices used to characterize ECGs have been shown to accurately predict occlusion myocardial infarction in the absence of ST-segment elevation.^34^ Studies by Kolk et al^35^ and Rogers et al^36^ used mathematical time-series features (eg, Fourier transform and autocorrelation) to describe ventricular monophasic action potentials and 12-lead ECG, respectively, in the context of SCD. Rogers et al^36^ trained an AI prediction model on this large set of features to predict the risk of ventricular arrhythmia in 42 patients with LVEF < 40% and CAD. During 3-year follow-up, this model reached a C index of 0.90. In a cohort of ICD recipients for primary prevention of SCD, this approach predicted appropriate ICD therapy with an area under the receiving operating characteristic curve (AUROC) of 0.68, mainly driven by Fourier coefficients corresponding to low frequencies in ECG.^35^ In contrast to manually engineered features, DL models are empirical—rather than hypothesis based—and able to automatically learn complex relationships directly from raw data. An ECG-AI model developed using data from 2 prospective community-based studies was used to predict SCD, with an AUROC of 0.82 in an external validation cohort over a follow-up period of 1.6 ± 2.1 years.^30^ In combination with clinical variables, the AUROC increased to 0.90, outperforming a conventional ECG risk score constructed using standard (expert-hypothesized) ECG parameters. Additional ECG-AI models predicted SCD in patients with heart failure, with an AUROC of ~0.66, surpassing guideline criteria for primary prevention ICD and traditional ECG parameters.^37,38^

Despite the promising results achieved by ECG-AI models (Table 1), it is crucial to critically weigh their advantages and limitations in comparison to established methods. Several ECG-AI models have been evaluated alongside traditional ECG parameters and demonstrated superior performance^30,38–40^; however, the field of ECG-AI has been characterized by low-quality models built on ad hoc data sets, lacking external validation, and trained on insufficient sample sizes.^28,41^ In recent years, more attention has been paid to ensuring rigorous scientific standards for AI model development, validation, and evaluation, which are essential for distinguishing between high-quality models and those of lesser quality.^42,43^ Moreover, a gap in performance between ECG-AI models in the general population compared to at-risk patients can be observed, which may be explained by the fact that patients with compromised cardiac function already have baseline abnormalities in their ECG. This might challenge models to find specific ECG signatures that indicate SCD risk amidst these preexisting abnormalities. Moreover, 12-lead ECG provides a snapshot of the electrical phenotype of a patient that may not fully reflect the electrical instability that predisposes SCD.^24^ Barker et al^44^ assessed the performance of a DL model trained to predict SCD using a 24-hour ECG recording, in contrast to 10-second ECG, which reached an AUROC of 0.80 for 1-year SCD risk prediction. These findings suggest that a more comprehensive reflection of electrical instability over time might uncover prognostic ECG changes identified by the network. In addition, ECG changes can manifest because of a plethora of underlying causes, likely affecting the accuracy of models when the prediction horizon lengthens.^28,39^ Previous AI models predicted ventricular arrhythmia and cardiac arrest within 24 hours, with AUROCs that ranged between 0.86 and 0.95,^31,32,45,46^ which dropped to an AUROC of 0.78 when predicting 72 hours before the event.^47^ Cha et al^48^ assessed the performance of a DL model across different prediction windows using intracardiac electrograms obtained from the ICD. This model predicted ventricular arrhythmia treated by the ICD 3 seconds before the onset, with an AUROC of 0.83, which dropped to AUROCs of 0.54–0.56 when the prediction range extended beyond 30 days.^48^ Last, rather than predicting SCD from ECG, these ECG-AI models may facilitate the early screening of structural heart disease and estimation of left ventricular function (eg, ejection fraction and left ventricular diastolic function and filling pressure) and predict genetic predispositions that increase SCD risk.^25,49–53^

## D. Imaging-AI for SCD prediction

SCD caused by ventricular tachyarrhythmias critically depends on an underlying vulnerable substrate, which is geometrically complex in architecture and challenging to characterize using ECG alone.^54^ Advances in cardiac imaging techniques have opened new ways of noninvasive identification and characterization of the relevant arrhythmic substrate, which may provide incremental prognostic value beyond LVEF and ECG alone.^54^ In particular, by means of late gadolinium enhancement (LGE), cardiac magnetic resonance (CMR) imaging can inform about the quantity and patterns of myocardial fibrosis. Earlier studies have provided robust evidence regarding the prognostic value of LGE on CMR imaging in patients with ischemic and nonischemic heart disease; however, there is a paucity of data on the use of LGE to guide primary prevention ICD treatment.^54^ In a substudy of the DANISH study, the presence of LGE was unable to identify patients in whom ICDs prolonged survival.^55^ Furthermore, the high prevalence (w20%) of clinically silent and otherwise unrecognized myocardial fibrosis in the general population suggests that the presence of LGE alone may be a nonspecific predictor of SCD.^56,57^ There are also technical limitations of LGE mapping: diffuse fibrosis detected through T1 mapping can increase the risk of ventricular arrhythmia even in patients without identifiable LGE–magnetic resonance imaging (MRI) regions.^58^

As with ECG-AI, feature extraction from cardiac imaging has become widely used to better differentiate between clinically neutral and proarrhythmogenic substrates. Prior studies have characterized these substrates using mathematical formulations such as entropy and global scar irregularity, interface area between fibrosis and surviving myocardium, and transmural extent and radiality of fibrosis (Table 2).^59–64^ In an imaging-AI approach by Okada et al,^64^ a patient-specific substrate spatial complexity profile was computed that reflected both the global irregularity of signal intensity and the 3-dimensional (3D) geometry of the left ventricle in patients with ischemic cardiomyopathy. By using AI to model the region of tissue transition that may contribute to an unidirectional conduction block and the formation of reentrant circuits, this complexity profile provided incremental prognostic information for arrhythmia prediction. Moreover, characteristics of the fibrotic microstructure in patients with CAD used as input to an ML classifier predicted ventricular arrhythmia risk, with an AUROC of 0.81 during cross-validation.^65^ Aside from tissue characteristics, an imaging-AI–based geometric score revealed left ventricular 3D morphology to be an independent predictor of ventricular arrhythmia.^60^

DL has shown impressive results on a wide range of computer vision tasks, automatically extracting clinically relevant imaging features from raw images that may not be easily discernible through traditional computational methods. The Survival Study of Cardiac Arrhythmia Risk (SSCAR) deep neural network was developed to predict per-patient cause-specific survival curves for sudden arrhythmic death in patients with ischemic heart disease by using stacks of shortaxis LGE-MRI slices. This model achieved a C index of 0.74 and an integrated Brier score of 0.14 in an independent test set, predicting over a 10-year interval.^66^ To address the criticism that such models are difficult-to-understand black boxes, a heatmap that visualized regions influencing the model performance highlighted that the model had learned patterns across the entire myocardial wall. Although the representations derived from LGE-CMR imaging may reflect an arrhythmic substrate, these may not indicate other mechanistic risk predictors such as cardiac chamber dimensions, regional myocardial deformation, or dispersion of mechanical activation.^67,68^ In order to include such mechanistic information, Krebs et al^69^ used a neural network to extract deep representations from cine CMR imaging in 350 patients with ischemic cardiomyopathy, which predicted ventricular arrhythmia with a C index of 0.69 over a median follow-up of 7.1 years. The Deep Arrhythmic Prevention in Dilated Cardiomyopathy (DARP-D) model integrated long-and short-axis cine CMR imaging and LGE-CMR imaging to predict major arrhythmic events in 154 patients with LVEF < 50% and CAD. The model predicted at 1 year with an AUROC of 0.84, which dropped to 0.53 at 8 years.^70^

## E. Multimodal-AI and digital twins

The onset of ventricular arrhythmias arises from a cardiac substrate, a transient trigger (eg, acute ischemia), and the modulating role of the autonomic nervous system.^11^ Most of the current SCD prediction models have exploited a single data modality; however, the potential of AI is theoretically maximized when multiple modalities are integrated to create a comprehensive characterization of the physiological state that improves downstream tasks.^71,72^ This may encompass anatomical image features of substrate and 3D geometry, variants from genome-wide association studies, conduction patterns, electrical physiology, or other modalities (Table 3). A recent multimodal-AI approach combined DL features from both ECG and LGE-MRI, alongside clinical patient data, to predict the 1-year risk of ventricular arrhythmia. Autoencoders extracted the deep representations from LGE-MRI and ECG, which were concatenated into a multimodal fusion embedding with clinical features. This fusion embedding was used as input for the DEEP-RISK ML model, achieving an AUROC of 0.84 in an independent cohort, which was higher than that of either component modality. The model performance was driven predominantly by features from LGE-MRI, but ECG features add incremental value.^73^ Furthermore, a recent multimodal network showed that neural networks are able to learn fundamental physiology, underscoring both ECG and CMR imaging, which were in turn associated with genetic variants.^74^ This cross-modal learning approach not only enhanced the representations obtained from a single modality but also enabled the imputation of challenging-to-acquire modalities, such as MRI, from more readily accessible ECG. Another approach to combining information from multiple sources is *digital twinning*, which aims to incorporate mechanistic and statistical models within a dynamic framework to provide a virtual replica of the heart.^75,76^ A personalized virtual heart that integrates cardiac imaging and electrophysiological properties has been found effective for the assessment of substrate complexity, guide VT ablation, and predict postablation arrhythmia recurrence.^77–79^ Similarly, the risk of SCD after myocardial infarction was predicted by evaluating VT inducibility within a virtual heart constructed using LGE-CMR imaging.^80^ O’Hara et al^78^ combined LGE and T1 mapping within a personalized heart model for patients with hypertrophic cardiomyopathy, and using virtual pacing, they assessed VT inducibility and arrhythmic risk during follow-up. In patients with arrhythmogenic right ventricular cardiomyopathy genotypes, a genotype-specific digital twin of the heart has been developed. Not only did the genotype-specific digital twin predict the VT circuit locations, but also the underlying VT mechanisms differed among arrhythmogenic right ventricular cardiomyopathy genotypes.^81^ Ultimately, these computational modeling approaches may provide a noninvasive means for personalized risk stratification and ablation strategies.

## F. Capturing the temporal dynamics: Telemonitoring and wearable devices

The risk of SCD is not static but fluctuates over time because of factors such as lifestyle, changes in medication, progression of underlying conditions, and acute cardiac events.^24^ This necessitates a dynamic predictive framework capable of updating predictions over time.^1^ For instance, it has been observed that ventricular arrhythmias are often preceded by ventricular ectopy, such as nonsustained VTs or frequent premature ventricular complexes.^11^ Continuous patient monitoring or frequent registration of ECG could be used to capture these precursors of ventricular arrhythmias.^82^ A recent study demonstrated superior performance of a dynamic AI prediction model that updated predictions when a new ECG recording was available, compared to a static model that used baseline information alone.^39^ Moreover, Wu et al^83^ used information from sequential CMR imaging and heart failure hospitalization during follow-up to update a baseline ML model for appropriate ICD therapy, which outperformed the static model. In addition, continuous monitoring through cardiac implanted devices and wearables yields a wealth of personalized data that could potentially reflect disease progression and aid in the early detection of an increased risk of SCD. Earlier studies showed high accuracy for the realtime prediction of ventricular arrhythmia (<30 days) using data captured by the ICD, including activity levels, thoracic impedance, atrial arrhythmia burden, and lead impedance.^84,85^ Furthermore, acute myocardial ischemia is among the leading cardiac causes of SCD and ventricular arrhythmia.^7^ Therefore, it is crucial to focus on individuals with these *clean* phenotypes,^11^ where a primary ischemic trigger induces fatal ventricular arrhythmia.

## G. Challenges to widespread use of AI for SCD risk stratification

### G.1. Definition and verification of SCD

Algorithms trained on an imperfect reference standard—such as poorly defined or unadjudicated outcomes—are likely to produce unreliable predictions.^86^ Hence, a uniform definition and verification of SCD status for each individual in the training data set is essential.^86^ Among the primary challenges in SCD are inconsistencies in definitions and lack of verification. SCD is defined as an unexpected death due to a cardiac cause occurring within an hour of the onset of symptoms or 24 hours of having been observed alive and symptom free.^87^ However, this definition does not distinguish between nonarrhythmic and arrhythmic causes of sudden death and may even include noncardiac causes that were falsely attributed as cardiac. The Postmortem Systematic Investigation of SCD (POST SCD) autopsy study showed that only half of the presumed SCDs, defined by conventional epidemiological criteria, were proven to be arrhythmic after postmortem investigation.^7^ Of course, ICDs are effective only in preventing arrhythmic cardiac causes. Future models should attempt to include broader ranges of physiological and demographic data in an attempt to better classify arrhythmic and nonarrhythmic causes of sudden death. In a recent study of emergency medical service–witnessed SCD, an AI model achieved moderate accuracy in distinguishing pulseless electrical activity from ventricular fibrillation on the basis of demographic characteristics, medical history and medication, and prearrest symptoms.^88^ Further improvements and validation of such a model may potentially result in a pragmatic alternative for labeling of SCD and sudden arrhythmic death cases. Moreover, in selected cohorts of patients with an implanted device, surrogates for sudden arrhythmic death have been used that include ventricular tachyarrhythmias lasting >30 seconds, with hemodynamic compromise, or treated by the ICD through shock or antitachycardia pacing. However, appropriate ICD therapy for ventricular arrhythmia does not necessarily equate to actual sudden death, as ventricular arrhythmic events that trigger ICD therapy might have naturally self-terminated.^89^ Studies thus need to differentiate between shocks that are appropriate and those that are truly necessary in order to use these as surrogates.

### G.2. Representativeness and validation

The accuracy of AI prediction models is expected to fluctuate over time and across different settings.^86^ It is essential to identify and address any potential biases that may have been introduced during the training process, in particular through validation in one or, preferably, multiple independent patient cohorts. For instance, models trained on historical data that do not reflect current medical practice, such as those developed before the introduction of novel pharmacological treatments of heart failure, may inadvertently be biased. Moreover, models developed using data derived from carefully selected cohorts or clinical repositories with specific phenotypes are less likely to produce accurate results in cohorts where the composition and risk of SCD is different (Figure 1A). This raises concerns about how representative these data sources are of real-world clinical practice and, consequently, the generalizability of models trained using these cohorts.^28,43^ In addition, variations in SCD risk among different groups, including those based on socioeconomic status, race, and ethnicity, could introduce bias and affect the performance of these models if such factors are not adequately learned by the model.^90,91^ Furthermore, training data sets may contain inherent biases, such as inconsistencies in ECG acquisition or imaging techniques, that could incentivize the model to learn hidden nonsemantic cues from the acquisition pathway.^92^ Finally, in low-risk subgroups the incidence of SCD (<0.1 per 100 patient-years) results in highly imbalanced data, which can lead to a bias toward overclassifying the majority group and underrecognition of the SCD cases. Arguably, to effectively capture the complex, nonlinear interactions between features that might predict SCD and reduce the risk of overfitting, data sets need to contain thousands of samples per class. Overall, collaboration across multiple sites is crucial to achieving adequately sized databases for model development and validation, regardless of whether through the creation of a centralized data repository or the implementation of federated learning.

### G.3. Explainability, interpretability, and ethical constraints

**Figure 3:**
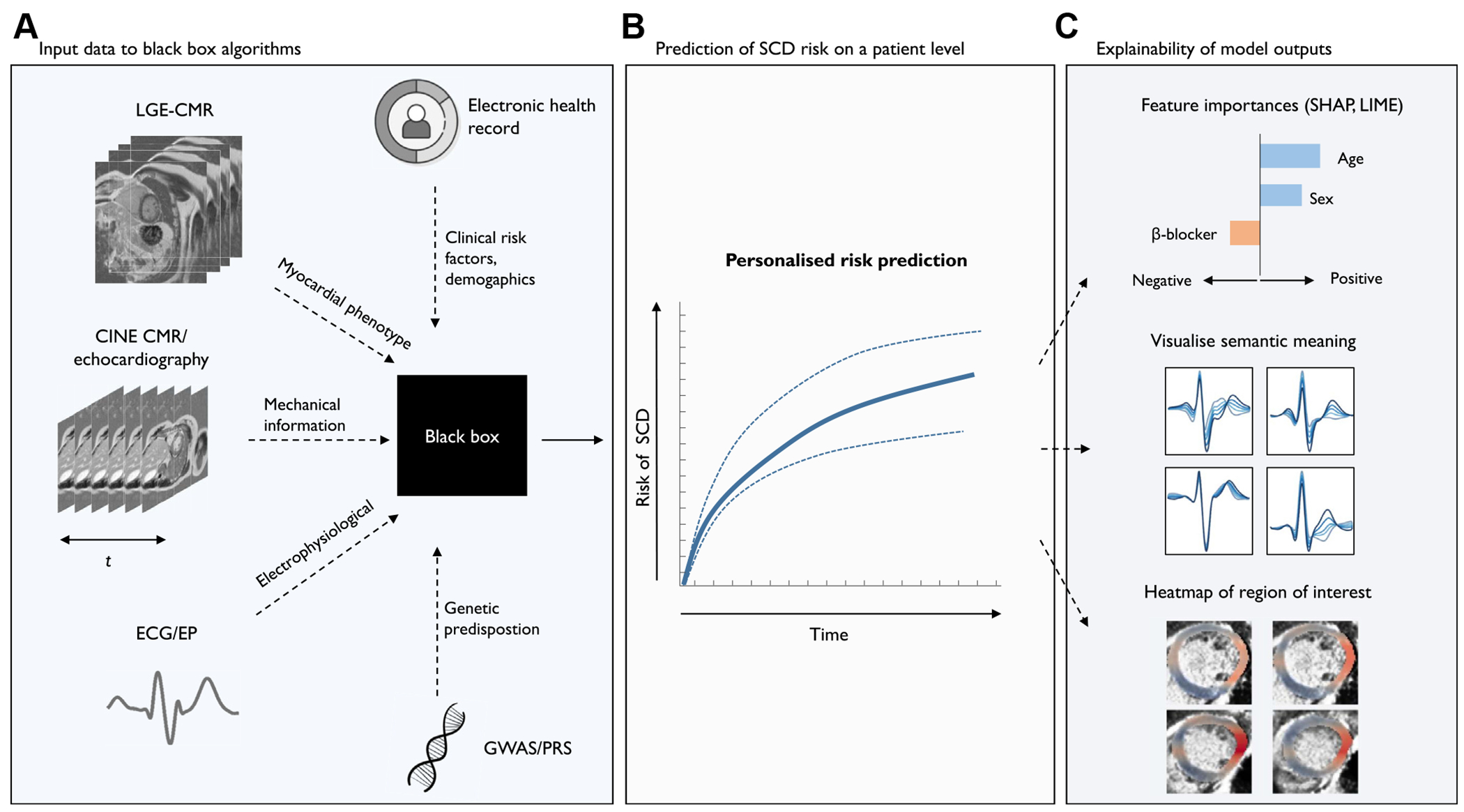
Multimodal AI has the potential to create a holistic characterization of an individual’s physiological state and leverage this for personalized risk prediction. The potential of AI is theoretically maximized when combining multiple modalities to form a comprehensive characterization of physiological states that improves downstream tasks. The state-of-the-art explainability techniques have focused on the development of the second (post hoc) model to explain the first black box model, which includes feature importance algorithms, factor traversal and latent space exploration, and gradient-based activation mapping. These methods approximate model explainability, but do not provide a complete understanding of why a specific prediction was made. AI = artificial intelligence; CMR = cardiac magnetic resonance; ECG = electrocardiography; EP = electrophysiology; GWAS = genome-wide association studies; LGE = late gadolinium enhancement; LIME = local interpretable model-agnostic explanations; PRS = polygenic risk score; SCD = sudden cardiac death; SHAP = shapley additive explanations.

DL models transform inputs through multiple nonlinear transformations, producing highly abstract and distributed representations of data. While these models function as *black boxes* where the internal workings are opaque, to reach the stage of clinical integration these models should be able to provide transparent explanations of their decisions and outputs. In particular, risk stratification for SCD and the potential consequence of withholding of a potential lifesaving prophylactic ICD urge physicians and patients to engage in shared decision making when deciding whether to implant an ICD.^93^ However, shared decision making demands the clinician and patient to receive sufficient and comprehensive information from the AI model for this process to be truly informed.^93^ Therefore, it has been suggested that explainability in AI-driven clinical decision making should be addressed at multiple levels: first, by defining what constitutes a sufficient explanation; second, by determining at which stage of the decision-making process explainability is required and how residual opacity is managed; and third, by assessing how these explanations are effectively integrated into the shared decision-making process.^94^ Rather than trying to create models that are inherently interpretable, explainable AI has focused on the development of the second (post hoc) model to explain the first *black box* model (Figure 3).^95^ However, none of these methods provide a complete understanding of *why* a specific prediction was made but serve as approximations of model explainability. Moreover, many of the complexities associated with shared decision making for SCD prevention go beyond what AI alone can manage, including patient preferences, values, and the impact on daily life.^93^ A rapidly growing set of solutions involves *interpretable* models, which map inputs or internal model representations to clinically or biologically meaningful features. One potential route toward inherently explainable ML is prototype-based models designed to mimic a clinician’s behavior by comparing new data to a set of representative examples, or *prototypes*. Such prototype-based models can indicate how strongly a prototypical pattern is present in an input, for instance, diffuse pattern of myocardial fibrosis on LGE-MRI or repolarization dynamics on ECG.^96^ A natural next step for AI models is to integrate interpretable and intervenable ML techniques to make models more intelligible.^97^ This might involve integrating human-model interactions or even embedding a *human in the loop* approach, where clinicians can interact with AI models to iteratively train on model errors.

### G.4. Clinical utility and integration

Although it is widely acknowledged that AI models should be designed to fit into real-world clinical workflows, substantial progress in translating these technologies into clinical practice has yet to be achieved.^98^ One of the main challenges in integrating a prediction algorithm for SCD into clinical settings is balancing the costs and resources required to collect the diagnostic inputs with their prognostic value. A serial testing strategy, which leverages the diagnostic modalities that are realistically available in a given target population, has been previously suggested for SCD risk stratification (Figure 1B). Such an approach could use established clinical models (eg, Seattle Proportional Risk Model (SPRM) and MADIT-ICD) and ECG-AI in low-risk groups, thereby prioritizing individuals for more detailed and resource-intensive screening, including imaging modalities or invasive testing. A serial testing strategy, although in a very specific population, was tested by van der Leur et al,^40^ who showed a 2-step method with an initial ECG-AI model to assess the need for further diagnostics in predicting SCD for patients with the PLN p.(Arg14del) variant to be more efficient and outperform a multimodal model. Of note, the potential synergy between established clinical risk factors and ECG-AI has demonstrated a significant improvement in accuracy, surpassing that of clinical factors and traditional ECG parameters combined with clinical factors.^23,30^ This underscores the potential of staged screening protocols for SCD risk stratification.

## Conclusion

Risk stratification for SCD remains one of the foremost challenges in modern medicine. AI provides novel means to model the perfect storm that leads to SCD, which involves the interaction between the vulnerable cardiac substrate with transient precipitating factors. In recent years, focus has shifted from traditional ML toward advanced deep neural networks and multimodal AI, which enable modeling of arrhythmic risk by learning a joint representation that integrates cardiac substrate (via cardiac imaging), genetic predisposition (from genetic repositories), and the electrophysiological state (through ECG and electrophysiology studies). However, translating these models to clinical practice faces several obstacles, including verification of SCD and ventricular arrhythmia, sparse availability of diagnostic information, insufficient sample size, unrepresentative patient cohorts, limited explainability, and lacking external validation.

## Figures and Tables

**Table 1:** Overview of ECG-AI studies for SCD prediction

Study (year)	Population	n	Signal	Features	Model	Follow-up	AUROC
Kolk et al (2024)^39^	ICD carriers	2,942	12-Lead 10-s ECG	DL features	VAE, RF-SLAM	90 d	0.74
Barker et al (2024)^44^	VA cohort	270	Holter ECG	DL features	CNN	1.6 y	0.80
van der Leur et al (2024)^40^	PLN p.(Arg14del) carriers	679	12-Lead ECG	DL features	VAE, Cox regression	5 y	0.79
Holmstrom et al (2024)^30^	General population	3,835	12-Lead ECG	DL features	CNN	2.0 y	0.89
Cha et al (2024)^48^	ICD carriers	13,516	EGM	DL features	CNN	30 d	0.55
Lee et al (2023)^32^	Hospitalized	4,821	Continuous ECG	HRV	LGBM	24 h	0.88
Shiraishi et al. (2023)^37^	Heart failure	2,559	12-Lead ECG	DL features	CNN	3 years	0.62
Kolk et al (2023)^35^	Primary prevention ICD, LVEF ≤ 35%	1,010	12-Lead ECG	Time-series characteristics	XGBoost	3 y	0.68
Sammani et al (2022)^38^	Dilated cardiomyopathy	695	12-Lead ECG	DL features	VAE, Cox regression	4.3 y	0.67
Rogers et al (2021)^36^	Coronary disease, LVEF ≤ 40%	42	Ventricular MAP	Time-series characteristics	SVM	3 y	0.90
Kwon et al (2020)^45^	Hospitalized	25,672	12-Lead ECG	DL features	CNN	24 h	0.95
Martinez-Alanis et al (2020)^99^	ICD carriers	91	EGM	HRV	SVM	1 min	0.68
Rodriguez et al (2019)^100^	Dilated cardiomyopathy	140	30-min ECG	HRV	SVM	2 y	0.95
Au-Yeung et al (2018)^33^	Primary prevention ICD, LVEF ≤ 35%	788	EGM	HRV	RF	5 min	81.0
Lee et al (2016)^31^	Hospitalized	15	Continuous ECG	HRV	ANN	1 h	0.75
Ong et al (2012)^47^	Hospitalized	925	5-min ECG	HRV	SVM	72 h	0.78

AI = artificial intelligence; ANN = artificial neural network; AUROC = area under the receiving operating characteristic curve; CNN = convolutional neural network; DL = deep learning; ECG = electrocardiography; EGM = intracardiac electrogram; HRV = heart rate variability; ICD = implantable cardioverter-defibrillator; LGBM = light gradient-boosting machine; LVEF = left ventricular ejection fraction; MAP = monophasic action potential; RF = random forest; SCD = sudden cardiac death; SLAM = survival, longitudinal, and multivariate; SVM = support vector machine; VA = ventricular arrhythmia; VAE = variational autoencoder; XGBoost = extreme gradient boosting.

**Table 2:** Overview of imaging-AI studies for SCD prediction

Study (year)	Population	n	Input	Features	Model	Follow-up	AUROC
Kolk et al (2024)^73^	Nonischemic cardiomyopathy, LVEF ≤ 45%	297	LGE-MRI and ECG	DL features	VAE, XGBoost	1 y	0.84
Coriano et al. (2024)^70^	Coronary artery disease, LVEF < 50%	154	LGE-MRI and cine	DL features	CNN	1 y	0.84
Zaidi et al. (2023)	Coronary artery disease	397	LGE-MRI	Shape-based microstructure features	Cox regression	6 y	0.81
Popescu et al (2022)^66^	Ischemic cardiomyopathy	269	LGE-MRI	DL features	CNN	10 y	0.72
Balaban et al (2022)^60^	Dilated cardiomyopathy	156	LGE-MRI	3D left ventricular shape	Cox regression	7.7 y	–
Krebs et al (2021)^69^	ICD recipients, prior MI, LVEF ≤ 35%	350	Cine	DL features	VAE, Cox regression	7.1 y	0.69
Okada et al (2020)^64^	Ischemic heart disease, LVEF ≤ 35%	122	LGE-MRI	Substrate spatial complexity	Cox regression	4 y	0.72
Wu et al. (2020)^83^	Primary prevention ICD, LVEF ≤ 35%,	382	LGE-MRI	Structural and functional indices	RF-SLAM	180 d	0.88

3D = 3-dimensional; AI = artificial intelligence; AUROC = area under the receiving operating characteristic curve; CNN = convolutional neural network; DL = deep learning; ECG = electrocardiography; ICD = implantable cardioverter-defibrillator; LGE-MRI = late gadolinium enhancement magnetic resonance imaging; LVEF = left ventricular ejection fraction; MI = myocardial infarction; RF = random forest; SCD = sudden cardiac death; SLAM = survival, longitudinal, and multivariate; VAE = variational autoencoder; XGBoost = extreme gradient boosting.

**Table 3:** Overview of studies using digital twins for SCD prediction

Study (year)	Population	No. of patients	Scan	Follow-up	Outcome
O’Hara et al (2022)^78^	Hypertrophic cardiomyopathy	26	LGE-MRI with postcontrast T1 maps	–	Patients with VA had an average of 2.10 ± 1.29 (P < .01) VAs induced as compared with 0.46 ± 0.77 in control patients
Shade et al (2021)^79^	Cardiac sarcoidosis	45	LGE-MRI and FDG-PET	1.7 y	Virtual heart model reached an AUROC of 0.75
Shade et al (2020)^101^	Tetralogy of Fallot	7	LGE-MRI	–	Simulations correctly identified patients who experienced clinical VT
Arvelo et al (2016)^77^	Prior MI and LVEF < 35%	41	LGE-MRI	6.8 y	Induced VA was significantly associated with the primary end point

AUROC = area under the receiving operating characteristic curve; FDG-PET = fluoro-deoxyglucose positron emission tomography; LGE-MRI = late gadolinium enhancement magnetic resonance imaging; LVEF = left ventricular ejection fraction; MI = myocardial infarction; SCD = sudden cardiac death; VA = ventricular arrhythmia; VT = ventricular tachycardia.
